# The establishment of a chemically defined serum-free culture system for human dental pulp stem cells

**DOI:** 10.1186/s13287-018-0928-8

**Published:** 2018-07-11

**Authors:** Jingyi Xiao, Dawei Yang, Qiwen Li, Weidong Tian, Weihua Guo

**Affiliations:** 1 State Key Laboratory of Oral Diseases & National Clinical Research Center for Oral Diseases & National Engineering, Chengdu, China; 2 Laboratory for Oral Regenerative Medicine, West China Hospital of Stomatology, Sichuan University, Chengdu, 610041 China; 3 Department of Oral and Maxillofacial Surgery, West China Hospital of Stomatology, Sichuan University, No.14, 3rd Section, Renmin South Road, Chengdu, 610041 People’s Republic of China; 4 Department of Pediatric Dentistry, West China School of Stomatology, Sichuan University, Chengdu, 610041 People’s Republic of China; 5 Department of Prosthodontics, West China Hospital of Stomatology, Sichuan University, Chengdu, 610041 China

**Keywords:** Essential 8 Medium, Dental pulp stem cells, Serum-free culture, Dental stem cell bank

## Abstract

**Background:**

The concept of establishing a dental stem cell (DSC) bank for oral and maxillofacial regeneration has become of great interest but it remains at a primitive stage. The routine application of serum-containing conditions for human DSC (hDSC) culture is in great controversy considering that the animal-originated serum can cause serious ethical concerns and lead to increasingly irrelevant variables, errors, and poor repeatability of experiment results. Thus, this study aimed to establish a safe, stable and efficient hDSC serum-free culturing system for future DSC bank usage.

**Methods:**

Dental pulp stem cells (DPSCs) from human permanent tooth pulp were isolated, expanded, passaged, and divided into two groups according to their culture conditions: group 1 was the serum-containing medium (SCM) group; and group 2 was the serum-free Essential 8 medium (E8) group. DPSCs were characterized first, followed by cell proliferation, pluripotency, and migration study in SCM and E8 medium.

**Results:**

Human DPSCs (hDPSCs) in E8 medium demonstrated greater proliferation, pluripotency, migration ability and less apoptosis. hDPSCs could be successfully induced to the adipogenic, osteogenic, neurogenic, and chondrogenic lineages in E8 group. Real-time polymerase chain reaction indicated that the expression of PPAR-γ, RUNX2, OCN and MAP-2 was higher in E8 group.

**Conclusions:**

Compared with serum-containing medium, E8 medium exhitibed higher ability in maintaining the cell proliferation, pluripotency, migration, and stability. This new serum-free culture environment might be applicable for hDSC culture in the future.

## Background

Human pluripotent stem cells (hPSCs) are clonogenic cells capable of self-renewal and multilineage differentiation. Over the past decades, hPSCs have shown tremendous potential for regenerative medicine, assisted reproductive technologies, and cell therapeutics [1]. Recently, Dever et al. [2] presented a CRISPER/Cas9 gene-editing system with homologous recombination at the HBB gene in hematopoietic stem cells and achieved 90% targeted integration of purified hematopoietic stem and progenitor cells. By sorting fetal human pancreatic α, β, and δ cells by staining for certain hormones, Blodgett et al. [3] demonstrated extraordinarily high levels of mRNA expression which might pave the way for new strategies for type 1 and type 2 diabetes treatment. Stem cells can also be applied for chronically injured organs. Karantalis and Hare [4] summarized the biology of mesenchymal stem cells and reviewed their wide utilization in cardiac tissue repair and regeneration therapy. Despite the tremendous achievements made over these decades, challenges still exist for taking clinical application of hPSCs to an industrialized scale and pharmaceutical grade production level [5], such as shortages of human stem cells available for research use [1], a low perfect histocompatibility match rate between potential recipients and transplant donors [6], and the lack of understanding of the immune response towards the majority of stem cells [7]. Lack of appropriate cryopreservation techniques and recovery methods also contribute to the barrier to the wide utilization of hPSCs [8]. Despite all the obstacles described above, the biggest concern is the culture condition.

Over the past decade, methods for hPSC culture have evolved rapidly to meet the urgent needs of drug discovery and regenerative medicine; however, many problems still need to be resolved, including the lack of specific applications of universally agreed standardized protocols and impurity or heterogeneity of cells [9]. Traditionally, the medium has included serum of either animal or human origin that was added for cell expansion. However, serum of human origin has raised ethical issues and there is a limited amount of collected human serum; therefore, culture medium with added products of animal origin, such as fetal bovine serum (FBS), has become a routine protocol. However, increasing evidence has indicated that FBS is a controversial ingredient owing to its risk of transmitting prion, zoonotic, or viral infections, and the xenogeneic compounds can trigger host immune responses, which are a potential and significant hazard. Another important concern is the batch-to-batch variety of the quality and protein concentration [10–12]. The continuous maintenance of undifferentiated stem cells over the long periods of culture is also essential [13]. Related research, including multiple culturing methods and ingredients, are emerging for the establishment of serum-free medium (SFM). The first attempt at culturing stem cells without using animal products dates back to 1976 when Hayashi and Sato [14] mixed four kinds of hormones (T3, TRH, transferrin, PTH) for stem cell incubation. Hirata et al. [15] demonstrated the sustained proliferation and expression of selected stem cell markers after culturing mouse DPSCs in serum-free media supplemented with a variety of growth factors. Bonnamain et al. [16] also reported successful expansion of human stem cells in a chemically defined serum-free culture medium with added growth factors. In the meantime, discoveries of signal pathways and multiple factor mechanisms in serum-free medium are showing steady progress, and a number of genes connected with the response have been unraveled, contributing to the use of hPSCs as engineering tools for therapeutic purposes [17–21].

Dental pulp stem cells (DPSCs) were firstly described as stem cells by Mooney et al. [22] in 1996. Gronthos and colleagues [23] reported the isolation and characterization of a stem cell population within the dental pulp. Because of the positive expressions of mesenchymal stem cell (MSC) markers such as STRO-1, CD13, CD24, Oct4, Nanog, and β2 integrin, the strong proliferation, self-renewal, and multiple differentiation ability [15, 24, 25], and easy access and convenient reservation [26], it is feasible to expect their application for dental regeneration, hard tissue engineering, and bio-root regeneration [27].

As dental tissues are an easily available source and the isolation of dental stem cells (DSCs) is a relatively easy and straightforward procedure [28], the concept of establishing a DSC bank has arisen. However, compared with the umbilical cord blood stem cell bank that has been well established for decades, the DSC bank is still at a very primitive stage. The term “tooth bank” was first raised in 1966 [29], but it was not until 2004 that the first commercial tooth bank was established at the National Hiroshima University in Japan as a venture company [30].

The culturing environment also plays a key role in the continuous maintenance of undifferentiated human DPSCs (hDPSCs) over the long term. However, most of the research and discoveries concerning media aimed at human embryonic stem cells (hESCs), and suitable SFM for hESCs might not be applicable for hDPSCs. Thus, despite all the effort and research devoted to this field, there is still no protocol for a well-defined, serum-free culturing condition for hDPSCs to maintain their proliferation capacity and differentiation potential.

E8 medium is a chemically defined, albumin-free medium created by the laboratory of James Thomson [31] designed for induced pluripotent stem cell (iPSC) culturing for both clinical applications and research use. E8 is a chemically defined medium containing Dulbecco’s modified Eagle’s medium (DMEM)/F12, 64 mg/l l-ascorbic acid-2-phosphate magnesium, 14 μg/l sodium selenium, 100 μg/l fibroblast growth factor (FGF)2, 19.4 mg/l insulin, 543 mg/l NaHCO_3_, and 10.7 mg/l transferrin, 2 μg/l transforming growth factor (TGF)β1 or 100 μg/l Nodal. Osmolarity of the medium was adjusted to 340 mOsm at pH 7.4, which simplified the quality control and reduced the financial cost. This simplified medium also gives a cleaner background for experiments on cell death, cell differentiation, and self-renewal, and significantly improves reprogramming efficiency. Thus far, E8 is a relatively mature medium and, furthermore, some components in E8 such as FGF2, TGFβ1, and insulin have been shown to be essential for the maintenance and growth of hDPSCs. This makes its use for analyzing the culture conditions of hDPSCs very practical.

In the present study, we expanded hDPSCs in vitro under E8 medium conditions and conventional serum-containing medium (SCM) as a control, and investigated the differences in cell characteristics, cell proliferation capacity, and cell pluripotency potential.

## Methods

### Cell culture in E8 and SCM

Extracted deciduous and wisdom teeth were obtained and checked for the viability of their pulp. The extracted teeth surfaces were cleaned and then cut up by sterilized hammers and cracked open to extract the dental pulp. Afterwards, the pulp was digested in a solution of 3 mg/ml collagenase type I for 0.5 h at 37 °C, and then treated by trypsin for 2 min. Cell suspensions were seeded in culture plates (Thermo, USA) and cultured in DMEM with 10% FBS (Hyclone, USA). After cells reached a concentration of 80%, they were cultured under separate conditions: in the E8 group, the cell suspensions were incubated at a cell density of 2 × 10^5^ cells per culture plate in albumin-free E8 culture medium (Stemcell, USA) containing chemically defined and concentration-determined multiple factors; in the SCM group, the cell suspensions were plated at the same cell density in normal medium containing DMEM and 5% FBS as a control. Passage (P)3 cells were employed for the following analyses.

### Colony-forming unit fibroblast (CFU-F) analysis

P3 hDPSCs were digested, suspended, and incubated at a density of 10^4^ cells in 12.5-cm plates. The culture medium was replaced every 3 days. Cells were fixed and labeled with toluidine blue for 40 min at room temperature at 10 days, and a random circle with a diameter of 30 mm was chosen from each plate and the formed colonies within the circles were counted (growing cells with a spindle shape, and colony with > 50 cells).

### Flow cytometric characteristic analysis

Approximately 5 × 10^5^ cells were incubated for flow cytometric analysis for 1 h at 4 °C with anti-CD29 (555,443, BD, 1:1000, phycoerythrin (PE)), CD31 (303,104, Biolegend, 1:1000, fluorescein isothiocyanate (FITC)), CD44 (555,478, BD, 1:1000, FITC), CD45 (555,482, BD, 1:1000, FITC), CD73 (550,257, BD, 1:1000, PE), CD90 (555,595, BD, 1:1000, FITC), CD105 (561,443, BD, 1:1000, FITC), and CD166 (559,263, BD, 1:1000, PE). FITC-conjugated or PE-conjugated isotype-matched immunoglobulins were used to examine nonspecific staining. Goat anti-mouse and goat anti-rat IgG-FITC (Santa Cruz) were applied as the secondary agent. FACS Caliber (Becton-Dickinson, CA, USA) was used for the analysis.

### Flow cytometric cell cycle analysis

Before flow cytometric cell cycle analysis, both E8 and SCM groups were starved in DMEM culture medium without FBS for at least 24 h. Cells were fixed with ethanol and dyed with propidium iodide (PI). Data were obtained through the FACS Caliber (Becton-Dickinson, CA, USA) and analyzed by the cell number percentages at different stages on the PI fluorescence histogram using FlowJo software.

### Flow cytometric cell apoptosis analysis

Cells (10 ml) were obtained at a density of 5 × 10^5^ cells/ml and centrifuged for 5 min and washed twice. Sedimentations were incubated for 20 min at 4 °C. The results were analyzed with FACS Caliber (Becton-Dickinson, CA, USA) at an excitation wavelength of 488 nm; 515 nm absorbance was used to detect FITC fluorescence and 560 nm absorbance was used to detect PI fluorescence.

### Cell proliferation analysis by CCK-8

Cells were digested and plated into 96-well plates (Thermo, USA) at a density of 2 × 10^3^ cells/well with culture conditions applied. After 24 h, 48 h, 72 h, 96 h, and 120 h of incubation, these cells were tested according to the Cell Counting Kit-8 (CCK-8, Dojindo Laboratories, Kumamoto, Japan) procedure. Averaged data were used to produce CCK-8 growing curves for visual observation.

### Bromodeoxyuridine (BrdU) cell proliferation assay

hDPSCs were incubated with 30 μl BrdU (Sigma, Germany) for 48 h. The solutions were then dispersed, the cells were fixed, DNA was denatured, and anti-BrdU antibody (MAB3424, Millipore, USA) was applied. The secondary antibody used was Alexa fluor 488 goat anti-mouse (A11001, Invitrogen, USA). Fluorescence microscopy was used to view the stained cells.

### Immunofluorescence

hDPSCs were subcultured (2 × 10^5^ cells/well) into six-chamber slides (Thermo, USA), fixed in 4% paraformaldehyde for 0.5 h, perforated with 0.5% Triton X-100 for 0.5 h, and then treated with 1% bovine serum albumin (BSA) for 1 h. Anti-OCT4 (D121072, Shenggong, China), anti-SOX2 (ab97959, Abcam, UK), anti-NANOG (sc-33,760, Santa Cruz, USA), anti-DMP1 (sc-6551, Santa Cruz, USA), anti-DSP1-H (sc-73,632. Santa Cruz, USA), anti-OCN (AP2002a x, Zen, China), anti-OPN (ab8448, Abcam, UK), anti-RUNX2 (ab76956, Abcam, UK), anti-BMP2 (sc-23,299, Santa Cruz, USA), and anti-P53 (ab26, Abcam, UK), and the secondary antibodies donkey anti-goat immunoglobulin G (A21432, Invitrogen, USA), Alexa fluor 555-conjugated goat anti-mouse immunoglobulin G (A21422, Invitrogen, USA), and goat anti-rabbit immunoglobulin G (A21428, Invitrogen, USA) were applied. Nuclei were stained with DAPI. The stained cells were observed under an Olympus inverted microscope.

### Western blot

Cells were digested with the Total Protein Extraction Kit (KeyGene, China) and centrifuged to collect cell supernatants. A BCA assay was used to determine the protein concentration. Proteins (30 μg) were separated on 10% sodium dodecyl sulphate-polyacrylamide gel and transferred to a PVDF membrane, and then blocked with 5% nonfat dry milk dissolved in TBST (Tris-buffered saline, 0.1% Tween-20). The primary antibodies anti-DMP1, anti-DSPP, anti-OPN, anti-ALP, anti-RUNX2, and GAPDH were used at dilutions of 1:1000 for 2 h at room temperature, and then washed with TBST twice on a TS rocker. Secondary antibodies were applied in the same way. Bands were monitored with an electrochemiluminescence system (GE, USA).

### Multidifferentiation in hDPSCs

hDPSCs were seeded at a density of 1 × 10^5^ into six-well plates with 80% concentration. The cells were separately cultured in osteogenic-inducing medium (100 nM dexamethasone (Sigma, USA), 50 mg/ml ascorbic acid (Sigma, USA), 10% FBS, 5 mM l-glycerophosphate (Sigma, USA)) for 15 days, adipogenic medium (alpha-minimum essential medium (MEM) supplemented with 2 mM insulin (Sigma, USA), 10% FBS, 10 nM dexamethasone (Sigma, USA), and 0.5 mM isobutylmethylxanthine (IBMX; Sigma, USA)) for 15 days, chondrogenic medium (6.25 mg/ml insulin, 50 μg/ml dexamethasone, 50 mg/ml ITS + Premix, 10 ng/ml TGF-β1, 100 μg/ml pyruvate, 40 μg/ml valine, 100 μg/ml penicillin, 100 μg/ml streptomycin, 10% FBS, and DMEM high-sugar culture medium) for 15 days, and neurogenic medium (200 mM butylated hydroxyanisole (Sigma, USA), 2% dimethyl sulfoxide (DMSO), 10 mM forskolin (Sigma, USA), 25 mM KCl (Kelong, China), 1 mM hydroxycortisone (Sigma, USA), 5 mg/ml insulin (Gibco, USA), 2 mM valporic acid (Sigma, USA) and 2 mM l-glutamine (Sigma, USA)) for 3 h. Afterwards, neurogenic-induced cells were observed with immunocytofluorescence for expression levels of the neural cell marker bIII-tubulin (Abcam, USA) and chondrogenic-induced cells with anti-col II (ab37,412, Abcam, UK). Other cells were fixed in 4% paraformaldehyde for 10 min and incubated in 0.1% alizarin red solution (Sigma, USA) in TriseHCl (pH 8.3) for osteogenic differentiation detection, or stained with 0.3% Oil Red O (Sigma, USA) solution for adipogenic differentiation detection.

### RNA extraction and real-time polymerase chain reaction (PCR)

Osteogenic, adipogenic, and neurogenic differentiation-induced P3 hDPSCs were chosen for the real time-PCR detection. All groups were induced for 7 days (except for neurogenic differentiation for 3 h) and then cultured for 7 days. RNAiso Plus (Takara, Japan) was used to extract total RNA from the cells in accordance with the manufacturer’s instructions. The complementary DNA (cDNA) synthesis was processed by the Revert Aid First Stand cDNA Synthesis Kit (Thermo Scientific, USA). The primer pairs for RUNX2, OCN, PPARγ, MAP-2, and GAPDH are shown in Table 1.

**Table 1:** Polymerase chain reaction primer design

Gene	Forward primer (5′–3′)	Reverse primer (5′–3′)
Runx2	GAAATGCCTCTGCTGTTATGAA	CCGTTATGGTCAAAGTGAAACTC
OCN	CCTCTCTCTGCTCACTCTGCTG	ACCTTACTGCCCTCCTGCTTG
PPARγ	TCCGAAGAACCATCCGATTGAA	CCACAGCAAGGCACTTCTGA
MAP-2	GTGAGTGCAGATGCTGAGGT	TGGCAATGGGACTGTGTACT
GAPDH	TATGACTCTACCCACGGCAAG	TACTCAGCACCAGCATCACC

### Cell migration capacity analysis

hDPSCs were digested and centrifuged at 80% confluence. Sedimentations were suspended with DMEM. Cell density was adjusted to 3 × 10^5^/ml and culturing medium was added into 6.5-mm transwell 24-well plates with a sterile 8.0-μm pore polycarbonate membrane (Costar, USA) at a volume of 600 μl for each well with culture conditions applied. Cells were seeded into the upper wells with 120 μl DMEM. After 12 h and 24 h of incubation, cells in the upper wells were removed and lower well cells were washed twice in phosphate-buffered saline (PBS) and stained with crystal violet for 10 min after being fixed in 4% paraformaldehyde for 15 min.

Marker pens were applied to draw straight lines on the back of the six-well plates at a distance of 0.5 cm. Cells were seeded into the six-well plates at a density of 1 × 10^5^/ml with culture conditions applied. After 3 days of incubation, pipette tips were used to scratch according to the marked line. Samples were inspected at 0 h, 24 h, and 48 h.

### Statistical analysis

Results are presented as the means ± standard deviations. Statistical analysis was performed using Students’ paired *t* test. Statistical significance was accepted at *p* < 0.05.

## Results

### Changes in cell morphology

**Fig. 1:**
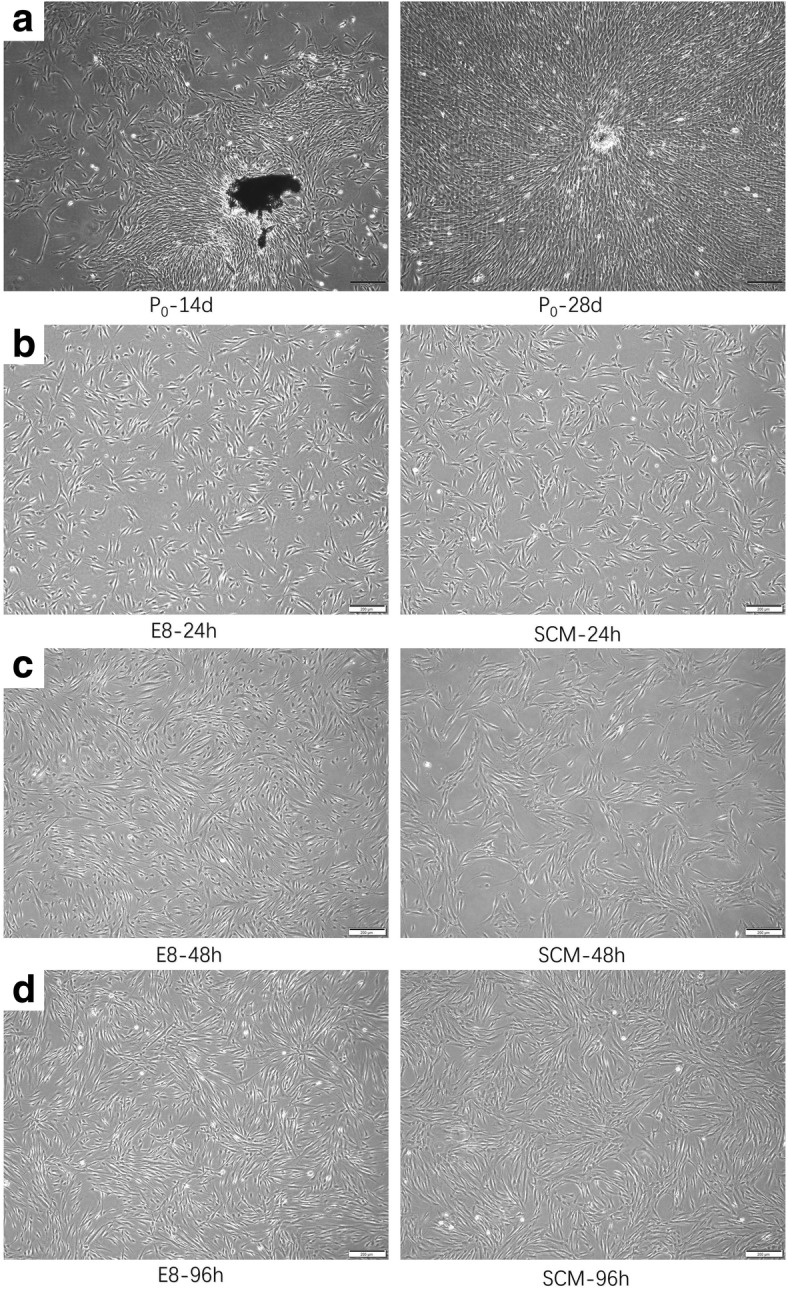
Cell morphology. **a** Images of primary culture for 14 d and 28 d. **b-d** Differences in cell morphology after culture in E8 (left) and serum-containing medium (right; SCM; DMEM + 5% FBS) for **b** 24 h, **c** 48 h, and **d** 96 h

Cells cultured in SCM proliferated sparsely in a single layer and demonstrated typical spindle and polygonal shapes. On the other hand, cells cultured in E8 tended to grow in close contact with one another and demonstrated more homogeneous shapes (Fig. 1). Cells cultured in E8 for 48 h and 96 h did not present differences in cell morphology.

### Identification of MSC surface markers

**Fig. 2:**
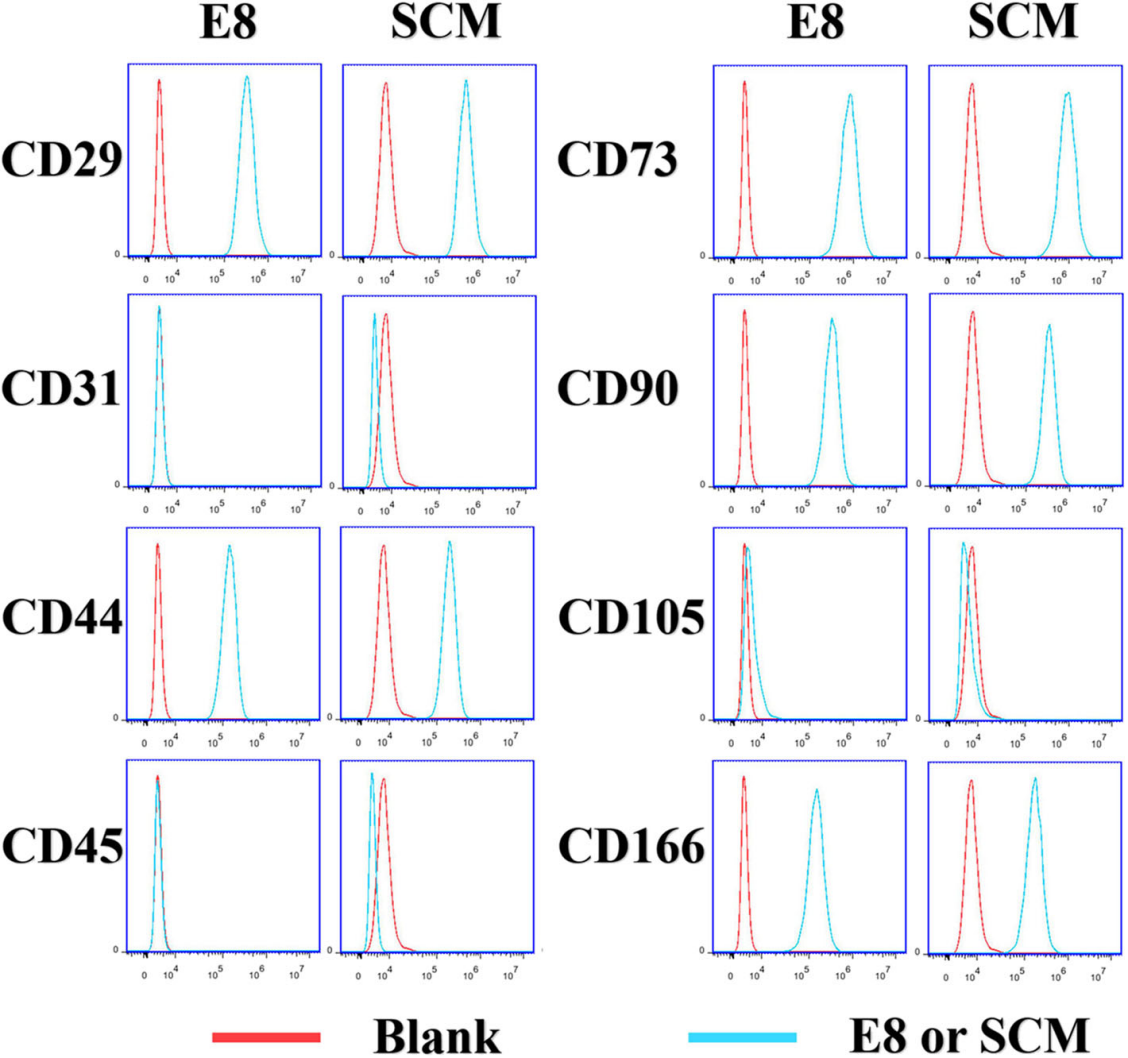
Characterization of hDPSCs surface markers by flow cytometry. The red curves are the blanks. The blue curves are the E8 or SCM.

Both the SCM group and the E8 group expressed high levels of CD29, CD44, CD73, CD90, and CD166, and did not express CD31, CD45, or CD105 (Fig. 2), which agreed with MSC surface marker expression and proved that the majority of these cells were DPSCs.

### E8 can promote hDPSC proliferation

**Fig. 3:**
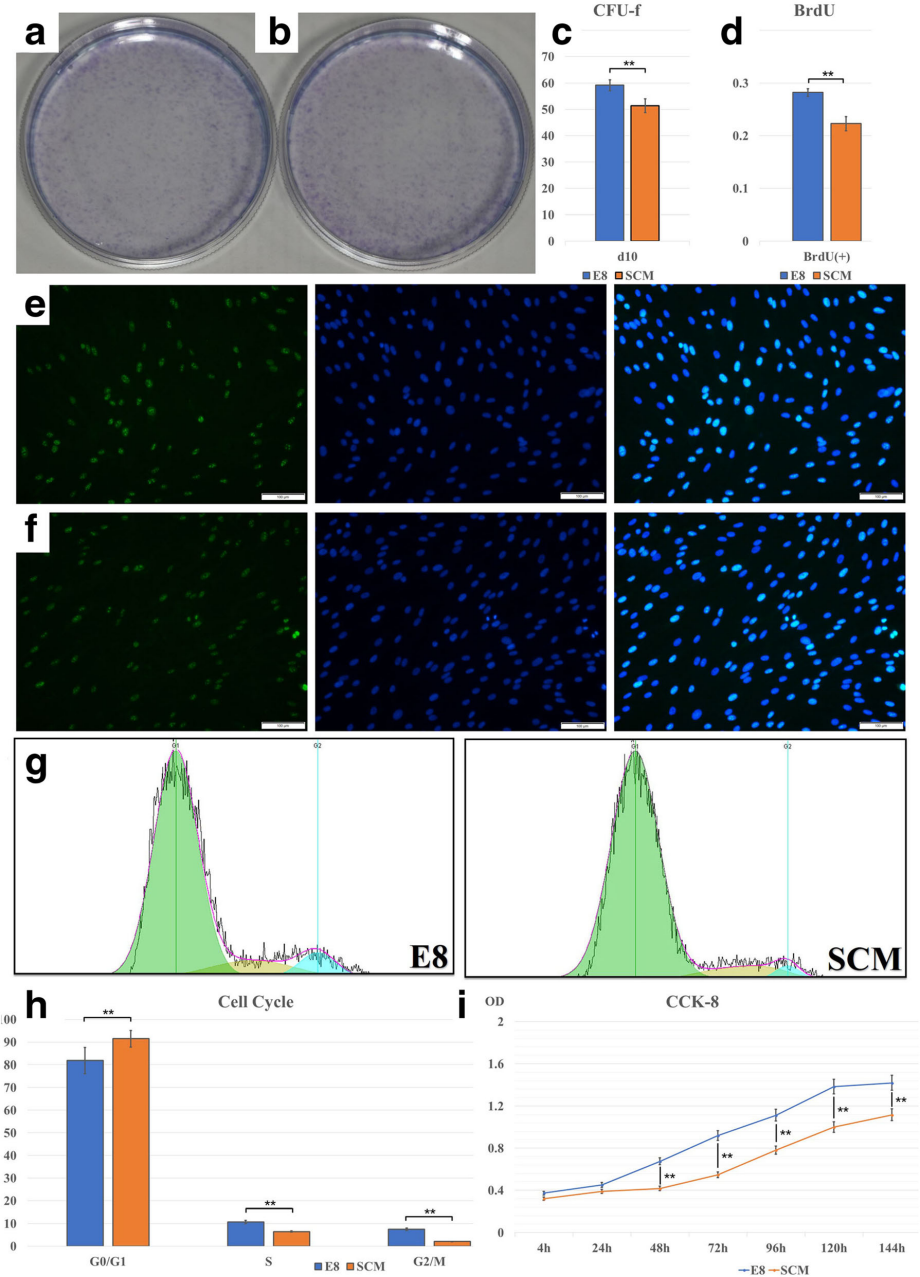
Colony-forming unit fibroblasts (CFU-F) of **a** serum-containing medium (SCM) and **b** E8. Statistical analysis of **c** CFU-F comparison (*n* = 5) and **d** bromodeoxyuridine (BrdU) proliferation assay (*n* = 5). BrdU fluorescence of hDPSCs in **e** E8 and **f** SCM. **g** Cell cycles were analyzed with FlowJo software. **h** Statistical analysis of the cell cycle (*n* = 5). **i** Cell proliferation analysis using the CCK-8 assay. The different optical density (OD) values are presented at 4 h, 24 h, 48 h, 72 h, 96 h, 120 h, and 6 days (*n* = 10). *p < 0.05, **p < 0.01

CFU-F results indicated that, at 10 days, a significant difference was observed between E8 and SCM (Fig. 3a–c) (*p* < 0.01). BrdU assay showed that, at 48 h, E8-cultured hDPSCs exhibited a stronger proliferation capacity with higher fluorescence labeling rate than culture with SCM (Fig. 3d–f) (*p* < 0.01). We used CCK-8 to analyze hDPSCs cultured for 4 h, 24 h, 48 h, 72 h, 96 h, 120 h, and 144 h. Data were obtained as average optical density (OD) values and a CCK-8 growth curve was produced (Fig. 3i) Statistical differences were observed between the E8 group and the SCM group at 24 h, 48 h, 72 h, and 96 h (*p* < 0.01).

**Fig. 4:**
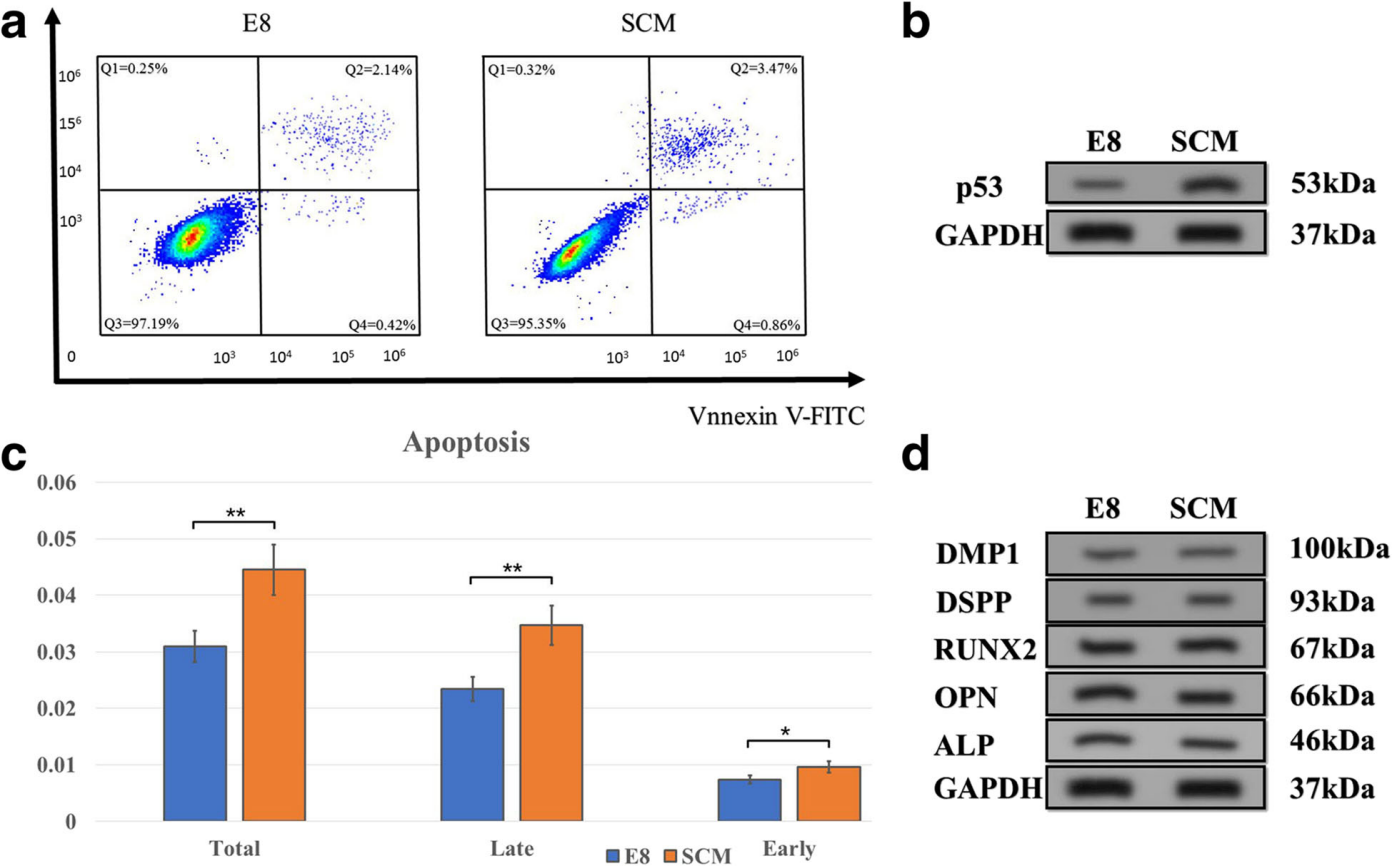
Cell apoptosis assay and Western blot. **a** Representative images of cell apoptosis from both E8 and serum-containing medium (SCM) groups. **b** Western blot images of cell apoptosis from both E8 and SCM groups. **c** Cell apoptosis comparison of the two groups (*n* = 5). **d** Western blot of DMP1 and DSPP (for odontogenic markers), OPN, RUNX2, and ALP (osteogenic markers), and GAPDH set as control. *p < 0.05, **p < 0.01

**Fig. 5:**
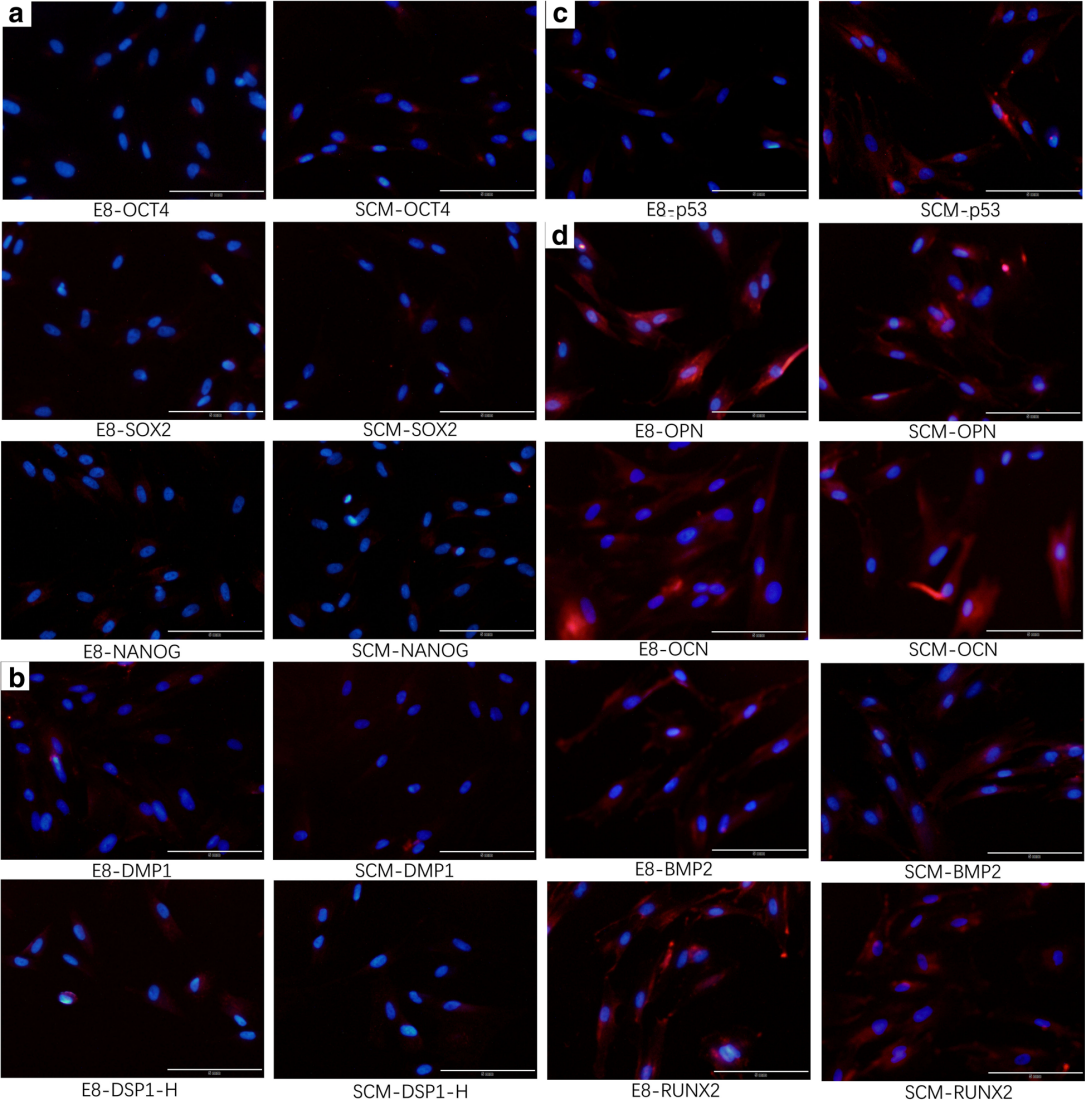
Immunofluorescence of hDPSCs. **a** OCT4, SOX2, and NANOG for cell pluripotency. **b** DMP1 and DSP1-H for odontogenic tendency. **c** p53 images of cell apoptosis from both the E8 and serum-containing medium (SCM) groups. **d** OCN, OPN, RUNX2, and BMP2 for the osteogenic tendency

To study why cell proliferation rate differed between E8 and SCM, we analyzed the cell cycle and apoptosis. Images captured by FlowJo software are presented in Fig. 3g. A significant difference was seen, and E8-cultured hDPSCs possessed fewer cell numbers in the G0/G1 ratio (*p* < 0.01) and higher numbers in the S ratio (*p* < 0.01) and G2/M ratio (*p* < 0.01) (Fig. 3h). Flow cytometry was used to analyze apoptosis, and the resultshowed difference between the SCM group and the E8 group regarding early (*p* < 0.05), late (*p* < 0.01), and total apoptosis (p < 0.01) (Fig. 4c). Images processed by FlowJo software are also presented in Fig. 4a. Western blotting and immunofluorescence also demonstrated that the apoptosis rate of hDPSCs in E8 group was lower than that in SCM group (Figs. 4b and 5c). Altogether, it can be deduced that the E8 medium increased the hDPSC proliferation rate through accelerating the cell splitting speed and decreasing the cell apoptosis rate.

### Expression of stem cell markers by immunofluorescence and Western blotting

Immunofluorescence was applied to analyze OCT4, SOX2, and NANOG for cell pluripotency, and a relatively low expression was obeserved in both E8 and SCM (Fig. 5a). Odontogenesis-related markers DMP1 and DSP1-H, also showed low expression (Fig. 5b). OCN, OPN, and RUNX2, analyzed for the osteogenic tendency, demonstrated high levels of expression, but BMP2 expression was lower in both groups (Fig. 5d). Western blot results also showed low expression in the odontogenic markers DMP1 and DSPP for both groups. For the osteogenic markers, Western blot results of OPN and RUNX2 showed expression at high levels, and ALP was expressed at low levels (Fig. 4d).

### Induced hDPSC differentiation analysis

**Fig. 6:**
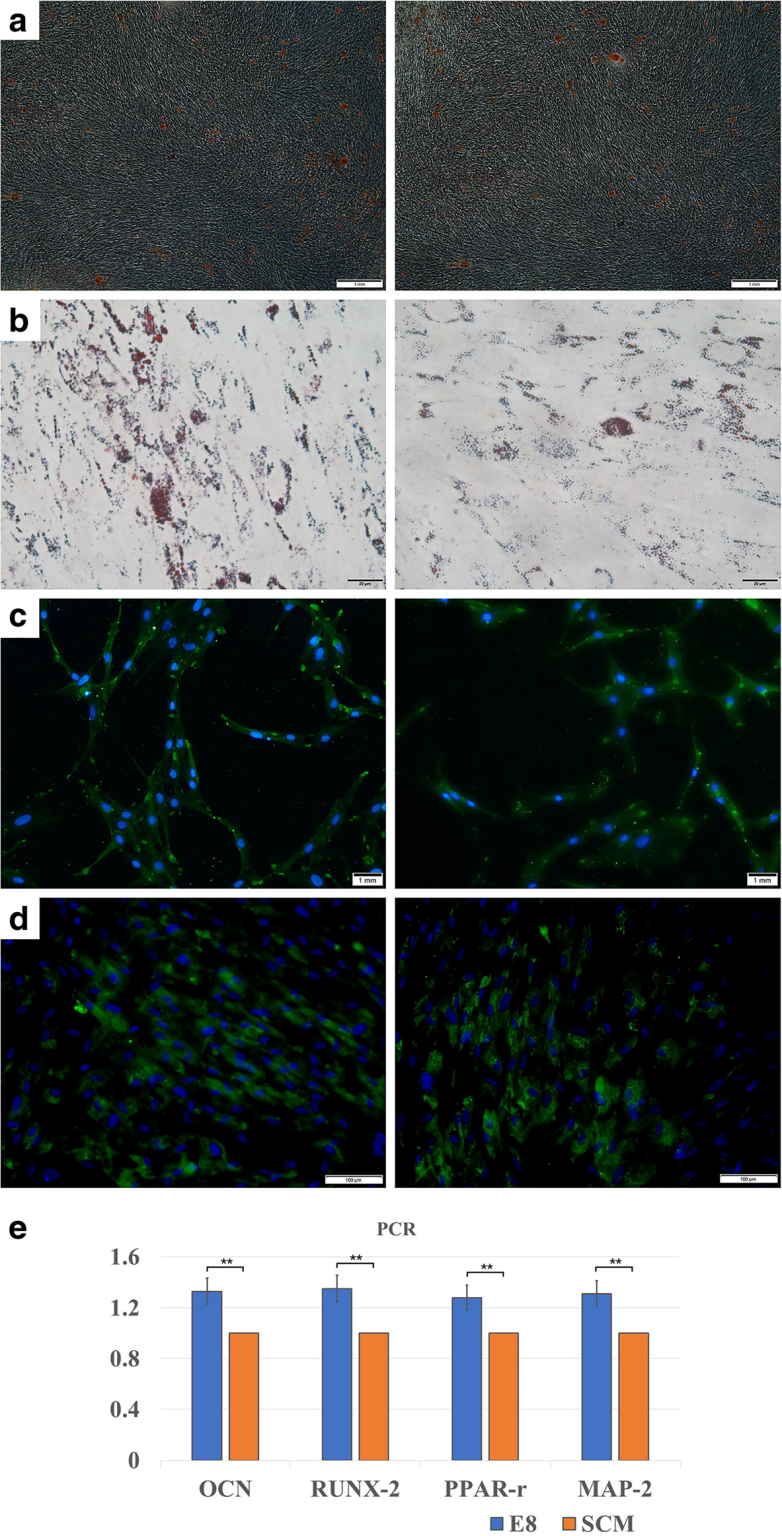
Multi-differentiation analysis of hDPSCs. E8(left); SCM(right) **a** hDPSCs cultured in osteogenic medium for 15 days. **b** hDPSCs cultured in adipogenic medium for 15 days. **c** hDPSCs cultured in neurogenic medium for 3 h. **d** hDPSCs cultured in chondrogenic medium for 15 days. **e** Real-time polymerase chain reaction (PCR) analysis of the multidifferentiation capacities of hDPSCs. Relative quantities were measured, and significantly higher levels of expressions of OCN and RUNX2 (for osteogenic differentiation), MAP-2 (for neurogenic differentiation), and PPAR-γ (for adipogenic differentiation) were detected in E8 compared with serum-containing medium (SCM) (*n* = 5). *p < 0.05, **p < 0.01

hDPSCs under E8 and SCM conditions at P3 were incubated and induced under different cultural conditions. In the osteogenic-inducing group at 15 days, hDPSCs formed mineralized nodules by alizarin red staining (Fig. 6a). After culture under adipogenic conditions for 15 days, hDPSCs formed lipid droplets when stained with Oil Red O (Fig. 6b). hDPSCs also exhibited green fluorescence under neurogenic and chondrogenic induction (Fig. 6c, d). Real-time PCR was used to further quantity the detection of the multidifferentiation capacities of hDPSCs. Interestingly, significantly higher levels of expression of OCN and RUNX2 (for the osteogenic differentiation test), MAP-2 (for neurogenic differentiation test), and PPAR- γ (for the adipogenic differentiation test) were detected in E8 (Fig. 6e) (*p* < 0.01).

### E8 promoted migration of hDPSCs

**Fig. 7:**
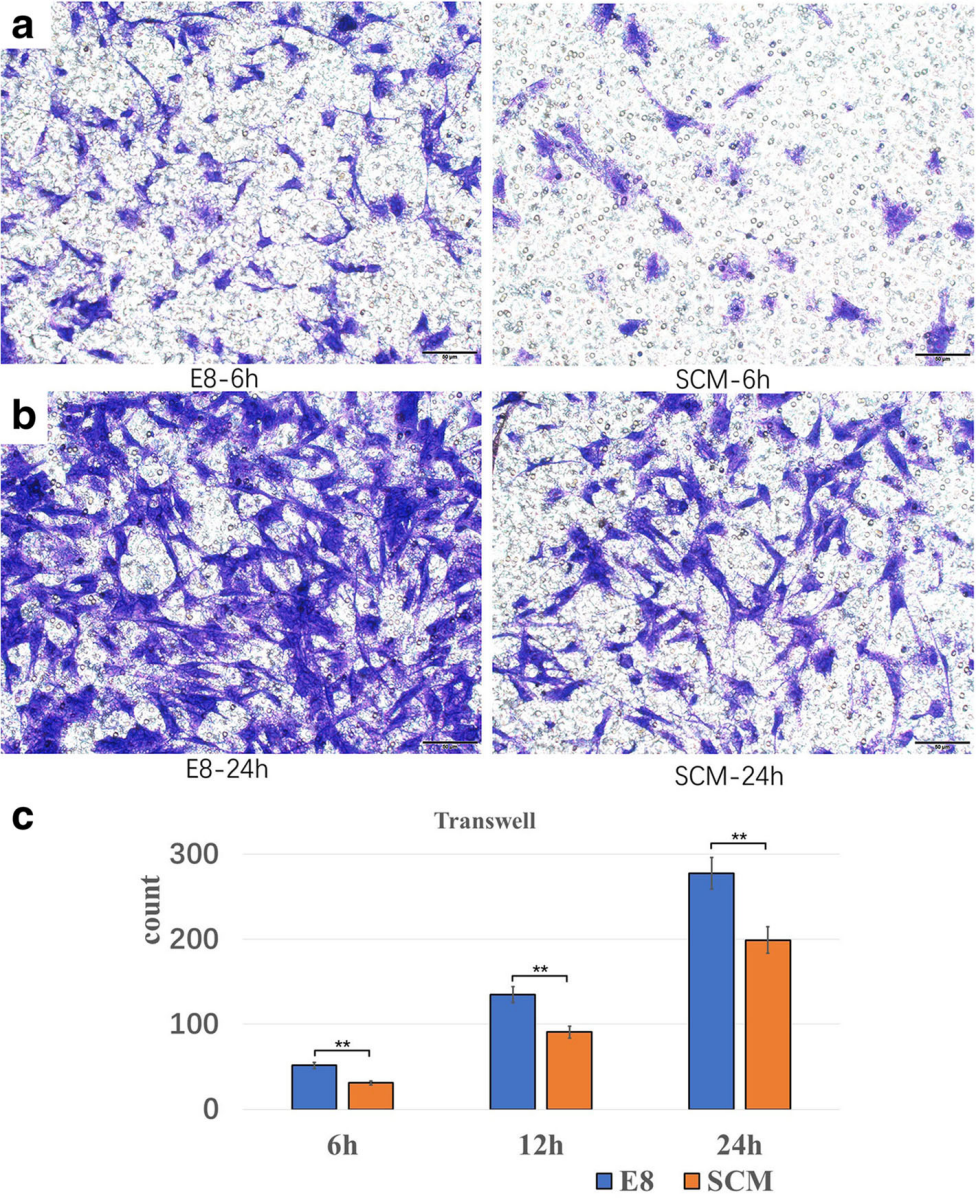
Migration capacity analysis by transwell for hDPSCs. Representative fields of images from transwell test are shown, with visual comparisons on migrated cell numbers between E8 and serum-containing medium (SCM) at **a** 6 h and **b** 24 h. **c** Statistical analysis of migrated cell numbers between E8 and SCM at 6 h, 12 h, and 24 h (*n* = 5). *p < 0.05, **p < 0.01

Using a transwell test, representative images of hDPSCs cultured in both media at 6 h and 24 h are demonstrated in Fig. 7a, b. E8 increased cell migration numbers at 6 h (*p* < 0.01), 12 h (*p* < 0.01), and 24 h (*p* < 0.01) (Fig. 7c).

**Fig. 8:**
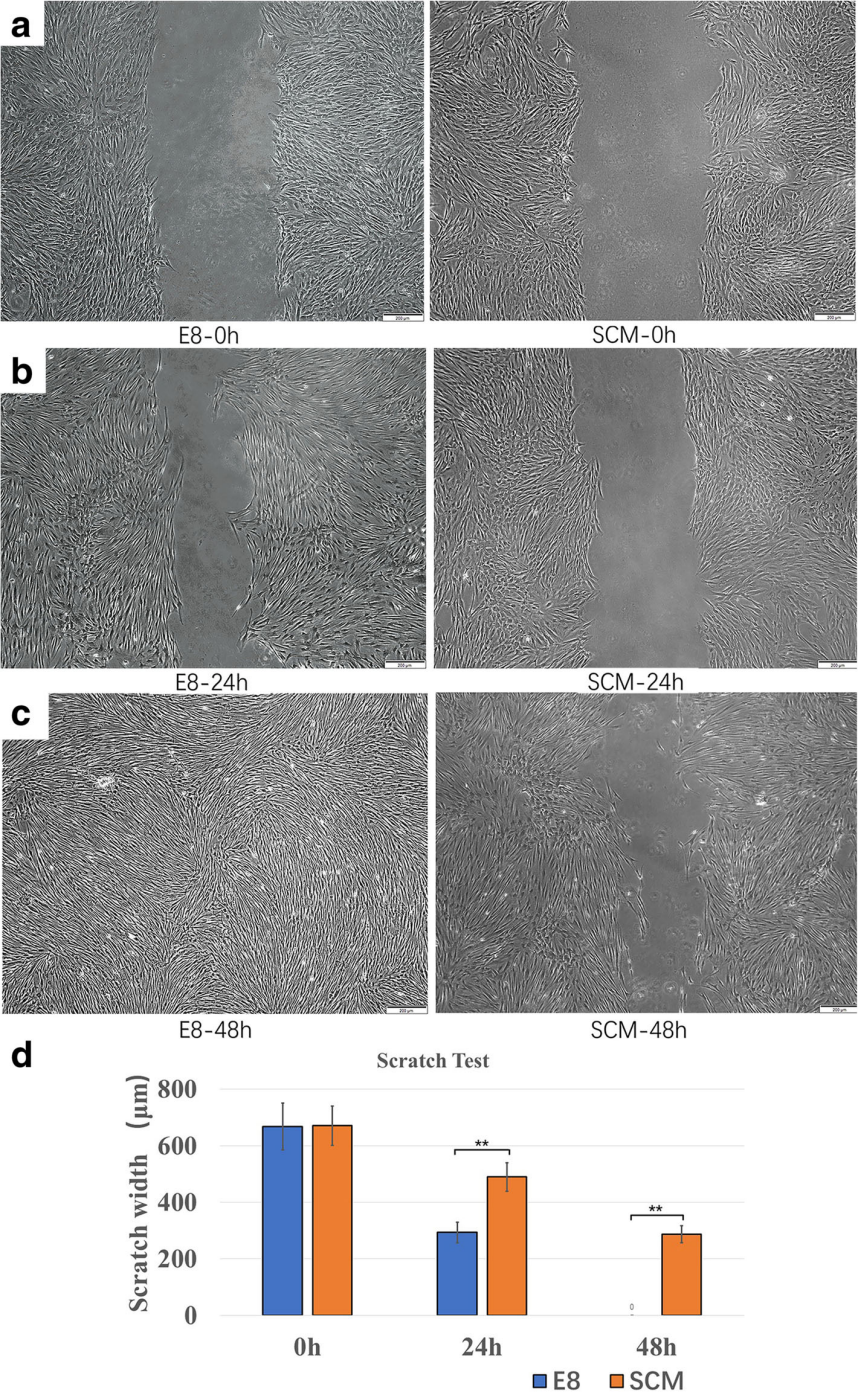
Migration analysis by scratch assay. Representative fields of images between hDPSCs cultured in E8 and serum-containing medium (SCM) are shown, with detections performed at **a** 0 h, **b** 24 h, and **c** 48 h under a microscope. **d** Statistical analysis of the comparison between E8 and SCM in the scratch length at 0 h, 24 h, and 48 h (*n* = 5). *p < 0.05, **p < 0.01

In the scratch assay, representative images of hDPSCs cultured in both media at 0 h, 24 h, and 48 h were captured under microscopy (Fig. 8a–c). The scratch width was the same at the start between the two groups (*p* > 0.05), and a clear motility difference was observed at 24 h (*p* < 0.01) and 48 h (*p* < 0.01) (Fig. 8d). A more obvious narrowing was observed at 24 h using E8. After 48 h of incubation, the gaps in the E8 group were invisible, while in the SCM group they were not.

## Discussions

Over the past decade, a substantial amount of research has focused interest on the need for the improvement of cell culture. In our study, hDPSCs were used to characterize and examine their properties. hDPSCs can be easily accessed and extracted from human third molars or deciduous teeth; however, as a type of highly differentiated stem cell, hDPSCs are commonly believed to be the least pluripotent among human DSCs. If hDPSCs could achieve satisfying proliferation and pluripotency performance in our serum-free culture environment, other human DSCs, such as periodontal ligament stem cells (PDLSCs), dental follicle stem cells (DFSCs) and dental epithelial stem cells (DESCs), might exhibit the same or better outcomes under the same culture conditions.

Although E8 was originally designed for iPSCs, the composition was shown to have multiple functions in stem cells, including in hDPSCs. FGF2 can regulate the Wnt signal pathway and its downstream signal via the PI3-K signal channel to maintain the undifferentiated state of hESCs and to maintain their self-renewal capacity [32, 33]. Ascorbic acid is a kind of water-soluble vitamin which plays a critical role in the formation of collagen that can improve the cell proliferation. Insulin can induce the absorbance of both glucose and amino acids [34], and selenite supplementation of culture media is capable of restoring the antioxidative capacity of bone marrow MSCs and reducing intracellular reactive oxygen species (ROS) production and stress-related generation of micronuclei [35]. Activin/Nodal signaling through Smad2/3 activation is necessary to maintain the pluripotent status of hESCs [36, 37]. Other growth factors including platelet-derived growth factor (PDGF), basic (b)FGF, and epidermal growth factor (EGF) also play a key role in maintaining the proliferation rate and pluripotency of stem cells [38, 39]. All the informed research into the functions of the signal pathways of growth factors gives us the foundation to use E8 medium in our experimental group. Additionally, E8 only contains eight essential factors [31] at a determined concentration, which is simple and convenient if researchers are willing to adjust the density of certain compositions, or to facilitate with other methods for further studies.

To eliminate variation, we used the same batch of hDPSCs for primary culture. In this way, although the cells were not purified, they can be considered to contain the same cell components and pluripotency. During CCK-8 analysis we detected that the OD value between E8 and SCM (both without cell culture) were different, so we used the difference in OD value between the media alone and the media cultured with cells to reduce the error caused by culture media. Hirata et al. [15] characterized and assessed cell proliferation and pluripotency under SFM conditions, but insulin-transferrin-selenium-X supplements alone were not enough, so they added embryotrophic factor to reach the same proliferation speed. Previous research has revealed that ascorbic acid, a kind of water-soluble vitamin which is a part of the composition of E8, plays a critical role in the formation of collagen, and thus enhances cell attachment [34]. This might explain the reason why the E8 group reached a high speed of expansion faster. Cell migration analysis showed that E8-cultured hDPSCs also had better mobility, indicating that our serum-free culture condition performed better in maintaining cytoactivity. Nevertheless, further studies should be performed to exploit the specific mechanism of this condition.

Previous studies demonstrated that hDPSCs present similar cell surface markers to MSCs [40] and, under certain conditions, hDPSCs can be induced to adipose tissue, osseous tissue, and nerve tissue [41]. The immunofluorescence and Western blot results suggested that no significant differences in pluripotency were observed, indicating that both groups maintained the same pluripotency under conditions without stimuli. However, induced differentiation results showed that hDPSCs cultured in E8 presented higher levels of expression of OCN, RUNX2, and PPAR- γ, indicating that in the case of irritation E8 is more efficient for maintaining multipotentiality towards osteogenic and adipogenic tissues for hDPSCs. Earlier studies displayed similar outcomes, and some also revealed the potential to be induced to other cell lineages. Harada et al. [42] compared cell proliferation, cell morphology, and gene expression change between SCM and SFM, but they did not detect induced differentiation. Ishkitiev et al. [26] successfully induced DPSCs to the hepatic lineage under SFM conditions. However, they did not induce these cells into other lineages. Okada et al. [43] also successfully induced hDPSCs to the hepatic lineage with hydrogen sulfide, and showed the same or higher performance. Ishkitiev et al. [44] induced hDPSCs to differentiate into the pancreatic cell lineage under serum-free conditions. As stem cells extracted from dental tissues, hDPSCs should demonstrate advantages in dentistry and bio-root regeneration in the near future. In 2010, Huang et al. [45] showed that DPSCs were capable of forming vascularized pulp/dentin-like tissue in an empty human root canal when seeded onto a polylactic-co-glycolic acid (PLGA) scaffold. This study, consistent with previous research, supported the possibility of using hDPSCs to establish patient-specific or industrialized-grade cell lines for stem cell therapy.

Al-Saqi et al. [46] applied Mesencult-XF as their serum-free medium to analyze its maintenance of pluripotency and growth towards adipose-derived MSCs. In a pre-experiment, we attempted to use Mesencult-XF to culture hDPSCs. Although cells cultured in Mesencult-XF displayed satisfying growth, we observed cell attachment problems; however, this phenomenon might be due to a difference in the applied culturing process. Furthermore, Mesencult-XF is relatively costly, and clinical cultures consume lots of media. Our work here, for the first time, applied E8 as a chemically defined, serum-free medium for hDPSC culture and analyzed its performance. However, most of our work was based on short-term culture comparison, and long-term culture data are still a requirement for further exploration. Although serum-free medium was applied during the cell culture and incubation, the use of FBS for the isolation of a primary cell culture is still indispensable and there are no statistical data suggesting how this application will affect cell proliferation and pluripotency [30].

## Conclusions

In conclusion, our study presents a novel application of the E8 medium in hDPSC culture, and shows that E8 is practical for the proliferation and differentiation of hDPSCs to reduce the effects of the problems caused by FBS as described above. E8 possesses a simple composition for the convenience of further downstream processing and improvement, which makes it practical to us as a primary culture medium for the establishment of DSC banking. Nevertheless, many challenges and obstacles still need to be resolved for its ultimate use in the clinic, and the mechanisms and functions of growth factors in maintaining hDSPC potency and proliferation should be fully analyzed and understood to achieve the optimum culture environment. More effective factors are needed, and deeper studies are still required, for improvement of the serum-free culture conditions. Other issues, such as the application of FBS in cell passaging and cryopreservation should also be resolved to establish a mature SFM culture system which can meet the DSC banking standards.

## Abbreviations

BrdU: Bromodeoxyuridine
CCK-8: Cell counting kit
CFU-F: Colony-forming unit fibroblast
DMEM: Dulbecco’s modified Eagle’s medium
DPSC: Dental pulp stem cell
DSC: Dental stem cell
FBS: Fetal bovine serum
FGF: Fibroblast growth factor
FITC: Fluorescein isothiocyanate
hDPSC: Human dental pulp stem cell
hESC: Human embryonic stem cell
hPSC: Human pluripotent stem cell
MSC: Mesenchymal stem cell
OD: Optical density
P: Passage
PCR: Polymerase chain reaction
PE: Phycoerythrin
PI: Propidium iodide
SCM: Serum-containing medium
SFM: Serum-free medium
TGF: Transforming growth factor
